# Distribution Pattern of Gymnosperms’ Richness in Nepal: Effect of Environmental Constrains along Elevational Gradients

**DOI:** 10.3390/plants9050625

**Published:** 2020-05-14

**Authors:** Bikram Pandey, Nirdesh Nepal, Salina Tripathi, Kaiwen Pan, Mohammed A. Dakhil, Arbindra Timilsina, Meta F. Justine, Saroj Koirala, Kamal B. Nepali

**Affiliations:** 1 CAS Key Laboratory of Mountain Ecological Restoration and Bio-Resource Utilization and Ecological Restoration Biodiversity Conservation Key Laboratory of Sichuan Province, Chengdu Institute of Biology, Chinese Academy of Sciences, Chengdu 610041, China; mohamed_dakhil@science.helwan.edu.eg (M.A.D.); francismeta@yahoo.co.uk (M.F.J.); 2 University of Chinese Academy of Sciences, Beijing 100049, China; nirdeshnepal44@gmail.com (N.N.); arbintms@sjziam.ac.cn (A.T.); sarojkoirala1@gmail.com (S.K.); 3 Institute of Mountain Hazard and Environment, Chinese Academy of Sciences, Chengdu 610041, China; 4 Department of Biology and Microbiology, South Dakota State University, Brookings, SD 57007, USA; salina.tripathi1994@gmail.com; 5 Botany and Microbiology Department, Faculty of Science, Helwan University, Cairo 11790, Egypt; 6 Key Laboratory of Agricultural Water Resources, Hebei Key Laboratory of Soil Ecology, Center for Agricultural Resources Research, Institute of Genetics and Developmental Biology, Chinese Academy of Sciences, Shijiazhuang 050021, China; 7 Ministry of Forest and Environment, Department of Plant Resources, National Botanical Garden, Godavari, Lalitpur 44709, Nepal; kamal_nepali26@yahoo.com

**Keywords:** biodiversity, energy-water, mid-elevation peak, physical-tolerance, species richness patterns

## Abstract

Understanding the pattern of species distribution and the underlying mechanism is essential for conservation planning. Several climatic variables determine the species diversity, and the dependency of species on climate motivates ecologists and bio-geographers to explain the richness patterns along with elevation and environmental correlates. We used interpolated elevational distribution data to examine the relative importance of climatic variables in determining the species richness pattern of 26 species of gymnosperms in the longest elevation gradients in the world. Thirteen environmental variables were divided into three predictors set representing each hypothesis model (energy-water, physical-tolerance, and climatic-seasonality); to explain the species richness pattern of gymnosperms along the elevational gradient. We performed generalized linear models and variation partitioning to evaluate the relevant role of environmental variables on species richness patterns. Our findings showed that the gymnosperms’ richness formed a hump-shaped distribution pattern. The individual effect of energy-water predictor set was identified as the primary determinant of species richness. While, the joint effects of energy-water and physical-tolerance predictors have explained highest variations in gymnosperm distribution. The multiple environmental indicators are essential drivers of species distribution and have direct implications in understanding the effect of climate change on the species richness pattern.

## 1. Introduction

The elevational gradient of species richness is an ecological phenomenon widely used in describing the patterns of species richness at small geographical areas or the landscape level [1]. Moreover, it was found that species richness pattern based on elevation gradient is one of the significant factors in determining the distribution of organisms in montane biota [2]. Elevation gradient, at its core, is not only a substantial factor that regulates the distribution of organisms in the mountains; findings have also reported that there is a strong variation in climatic conditions along elevational gradients [3,4]. The fact that makes mountains an ideal area for investigating the species richness pattern is the variation in the environment and topography within a small geographical region [1,3,5,6,7]. Climatic components and the topography vary along the elevation that directly or indirectly regulates the species richness in both plants and animals [6,8,9]. Due to that, the elevation gradient at present is prioritized to expose the mechanisms that shape the diversity of species. The most common form of species richness pattern along elevational gradients as explained by [6] (pp. 200–205) can be grouped into three basic types namely a monotonous decline in species richness with increasing elevation [1,10,11,12,13,14], a linear increase in species richness [5,10,11,12,15,16], and a peak at intermediate elevation forming a humped relationship. Most studies conducted in the mountains around the globe have reported the hump-shape or the high number of species at the mid-elevation pattern [2,8,10,11,15,17,18,19,20,21,22,23,24,25]. These species richness patterns were well studied in various plant groups such as- lichen [18], bryophytes [26], and other vascular plants [1,20,24] (for example- pteridophytes [20], orchid [17], threatened plants [13], endemic plant taxa [14], seeded plants [25]) along elevation gradients in the Himalayan including Nepal. Gymnosperms are taxonomically treated as vascular plants; however, the phenology, morphology, habitat, and diversity of gymnosperms are different from angiosperms and ferns which are also considered as vascular plants [27]. Gymnosperms are non-flowering, naked seeded and slow growing plants mostly adapted to xerophytic habitat. They are characterized by the presence of needle or scale like leaves [27]. Therefore, the species richness pattern of gymnosperms should be studied separately. Despite the effort from ecologists to describe the richness patterns at the world’s highest elevation gradient, a coherent understanding of the processes controlling gymnosperms biodiversity has yet to emerge. 

Environmental variables are correlated with elevation, where the climate is considered as the most potent factor that drives diversity and distribution patterns of plants and animals along elevation gradients [9,14]. In areas with variation in climatic conditions caused by elevation and topography, species richness is high [3]. Therefore, understanding the processes that shape patterns of species richness of plants and animals along elevation gradients are essential in determining the consequences of climate change on local biota. To explore the species richness pattern and factors that governs it, community ecologists have put forward various hypotheses considering elevation gradient and environmental variables. Temperature, precipitation, humidity, and available solar radiation are such environmental variables that were hypothesized to be important factors responsible for the distribution of plants on the mountains [5,18,20,21]. As no single factor can explain the diversity within and among taxa, more than one hypothesis must be considered to explain the patterns in species richness [6]. Thus, in this study, we evaluated the role of energy and water, physical tolerance, and climatic seasonality in explaining the species richness patterns of gymnosperms along elevation gradients in Nepal. “Energy-water hypothesis” is the most common and highly discussed hypothesis that explains species richness patterns of a taxon [28,29]. The relationship between species richness, energy and water has been previously studied in plant taxa [16,20,30]. This hypothesis is based on the availability of water and energy to the plants. The energy-water hypothesis is highly significant in defining the species richness patterns along elevation gradients [7,30]. The higher the available water and energy, the higher is the diversity of species in an area [29]. There is a strong influence of water availability on species at low elevation, while the availability of sufficient energy favors the diversity of species at a higher elevation [25]. However, according to the “physical tolerance hypothesis,” only small groups of species can withstand harsh climatic conditions like extreme cold and aridity [31]. Plants growing in the tropical regions are expected to experience physiological stress due to severe climatic seasons. Numerous relative studies have also explained the species richness pattern supporting the physical tolerance hypothesis [30,32,33]. It is well-established that plants, on the one hand, cannot survive in an extreme cold or hot environment and, on the other hand, cannot withstand aridity. According to “climatic seasonality hypothesis”, stable climatic conditions support species richness. The climatic seasonality is also considered to promote the co-existence of species through increased niche opportunities [30,34,35]. 

The mountains provide ideal conditions to explore the relative role of temperature, water and their variations [25,28,36]. Thus, the most extended mountain range, the Himalaya, is an appropriate area to investigate the species richness patterns along elevation gradients. The Himalaya is a chain of mountains and the youngest mountain range on earth, initiated by the collision between the Indian plate and Eurasia between 45–35 million years. The collision caused the up-rise of the Himalaya that extent over 2450 km long (east-west) and 200–350 km wide (north-south) from Mount Nanga Parbat (8125 m above sea level) in the northwest extremity to Mount Namche Barwa (7756 m) at the southeast end within the geographical territories of Pakistan, China, India, Nepal and Bhutan [37]. The approximate 900 km east to west expansion of Nepal runs through the heart of the Himalayan regions and is separated by the river Mechi and the Mahakali in the east and west, respectively. It has the highest mountain in the world and one of the longest bioclimatic elevation gradients [37]. The Himalayan region is also a biological hotspot of floral and faunal diversity. The rich biodiversity of Nepal is a result of a complex topography and climatic heterogeneity within small geographic area [38]. The climate of a country ranges from a tropical zone to a zone of permanent frost within 150 km and elevation ranges between 60 m and 8848 m above sea level (i.e., Mount Everest, the highest mountain peak in the world) [37]. Climatic and topographic variations arguably made this mountainous region an excellent place for studying the species richness patterns and provide a data set to use in examining the driving factors that may affect the richness patterns of plants and animals including gymnosperms. 

Therefore, in this study, we examine the relative importance of aforementioned climatic affinities that could potentially contribute to the distribution of gymnosperms in Nepal along the elevation gradient. The main objectives of this study are - (i) to describe the species richness pattern of gymnosperms along elevation gradient in Nepal, and (ii) to assess the explanatory power of energy-water, physical tolerance and climatic seasonality in explaining the distribution of gymnosperms along the elevation gradients. To our knowledge, this is the first specific study to analyze the species richness pattern of gymnosperms along an elevation gradient in the Himalaya. Given that gymnosperms are continuously exposed to extreme climatic conditions of temperature and precipitation [39], studying the extremities based on a physical basis is the primary goal of our study. Due to climate change, the atmospheric temperature of Nepal has been increasing at a higher rate than the global average [39]. This change has been more distinct at higher elevations which might affect the biological process of species including the range shift [39]. Thus, our findings may allow conservation biologists to tap into the existing knowledge regarding environmental drivers of species diversity of gymnosperms. It helps conservationists reach decisions about how best to manage nature reserves, to encourage and hold onto high levels of species richness. Hence, documentation of elevational patterns and distribution of gymnosperms along with understanding underlying causes are essential either to get insights on diversity gradients or to formulate conservation programs in the face of a recent global change.

## 2. Results

**Figure 1:**
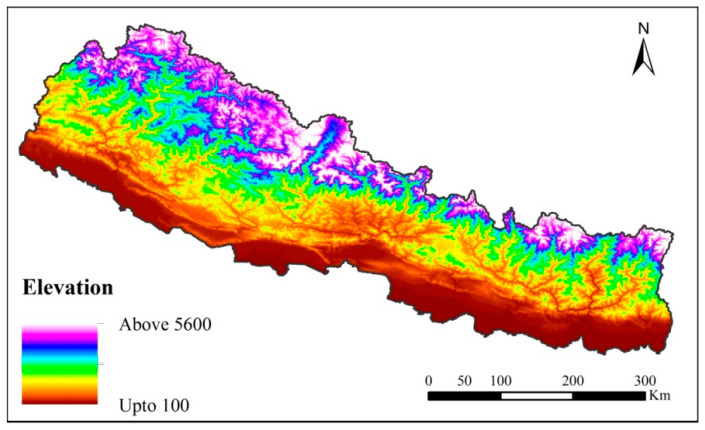
Map of Nepal showing 100 m elevational gradients.

**Figure 2:**
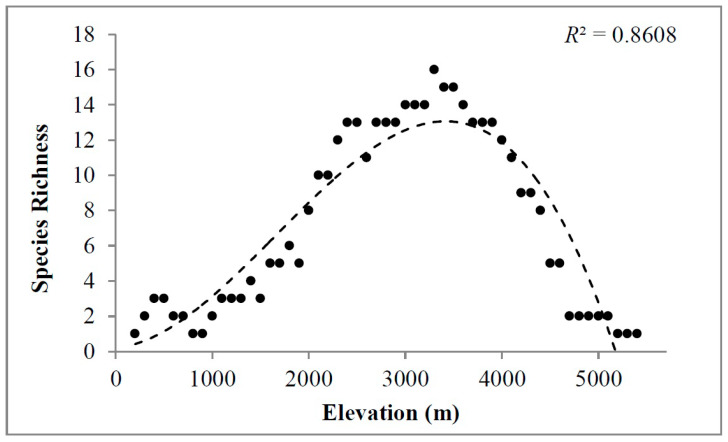
Species richness of gymnosperms in Nepal (solid dots) with the highest fit of the polynomial curve (dash line). *R*^2^ represents the explanatory power of the regression model significance at *p* < 0.005.

There was an abrupt increase in elevation in the country from south to north (Figure 1) and a total of 26 gymnosperm species were reported to distribute along Nepal Himalaya between 201–5400 m elevations. The highest recorded number of species (S = 16) was at 3300 m, and the pattern of species richness of gymnosperms was decidedly unimodal, forming a hump-shaped distribution pattern (*R*^2^ = 0.86) (Figure 2). As was apparent from Figure S1 of Supplementary Materials, the environmental predictors also vary along the elevation gradients. In general, all the environmental variables showed a monotonic decrease along the elevation gradients.

The generalized linear model showed that energy-water sets of variables were highly significant and showed higher explanatory power than the physical tolerance and climatic seasonality predictor sets (Table 1). The result showed that the species richness-energy and water relationship was stronger (*R*^2^_adj_% = 77.93%) than the climatic seasonality and physical tolerance in Nepal. The energy-water model had the best fit with high significance (*p* < 0.001) and the lowest Akaike’s information criterion (AIC) (219.9). The climatic seasonality model followed the energy-water model and explained 46.80% variance, while the model with the physical tolerance predictor sets provide the poorest fit, determining 38.64% variance in richness pattern of gymnosperms species. Both physical tolerance and climatic seasonality predictor sets showed a significant negative relationship with species richness (Table 1). 

**Figure 3:**
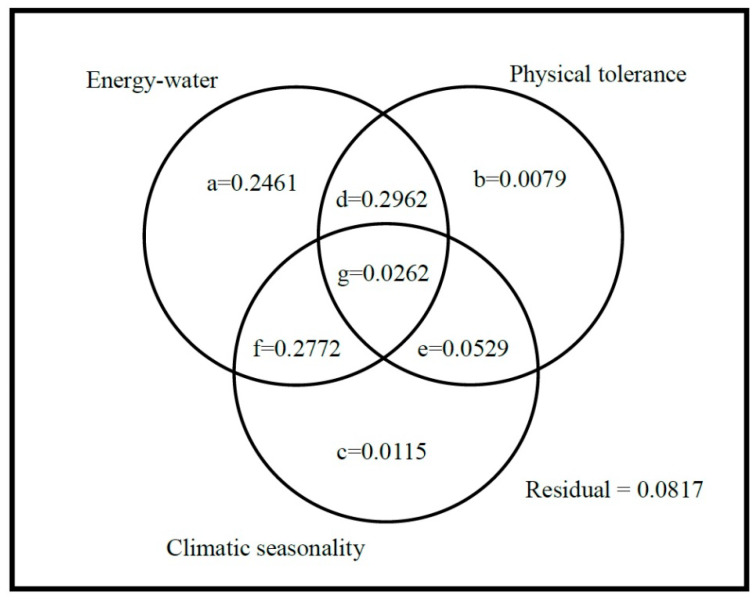
Results of variation partitioning between species richness and predictor sets. Each letter in the Venn diagram represents a fraction of variation partitioning analysis (the letter representing the fractions are mentioned in Section 4.4).

The result from variation partitioning of gymnosperms’ species richness showed that the variation explained by all three predictor sets was 91.82% and the remaining 8.18% was residual. The highest variation was accounted by the independent fraction of the energy-water predictor set, explaining 24.62% variation in species richness. The individual fraction of physical tolerance (7.9%) followed energy-water while the climatic seasonality variable set (1.15%) explained the least variation in species richness of gymnosperms (Figure 3 and Table S1 Supplement Materials). Moreover, the combined effect of energy-water and physical tolerance variables explained 29.62% variation, while the energy-water and climatic seasonality explained 27.72% variation in richness pattern of gymnosperms along elevation gradients (Figure 3 and Table S1).

## 3. Discussion

The gymnosperm richness in Nepal showed a significantly high number of species at 3300 m, forming a “humped-shaped” pattern. Such hump-shaped patterns were common in mountainous regions of the world including Nepal. Previous studies have also confirmed the unimodal pattern of species richness in different taxonomic groups of plants and animals [2,10,11,13,15,17,18,19,20,21,22,23,24,25,40]. Similar findings in the Himalayan region reinforce our findings [13,17,20,25,36]. All our results corroborate previous findings and support that species richness pattern of any taxa in mountain gradients is determined by environmental variables [7,20], however, this is the first comprehensive work that prioritized the distribution of gymnosperms along elevation gradients in Nepal. The elevation range between 2400 m and 3900 m above sea level lies within the temperate and sub-alpine zone of Nepal, characterized by the moderate and favorable climatic conditions for the distribution of gymnosperms [38]. Understanding the mechanism that determines the species richness pattern based on elevational gradients is essential for the conservation of gymnosperm species [38]. Thus, (i) the present study identifies the importance of environmental factors in determining the species richness pattern of gymnosperms in Nepal along the elevation gradients, and (ii) elevational gradients of species distribution will be a baseline for comparing the range shift of a population and prioritized in the conservation.

We found that energy-water predictor set best explained the richness pattern of gymnosperms in Nepal. The energy and water variables used in this study were calculated using latent heat from solar radiation, precipitation and soil water availability to plant, that in turn help in growth and development of plants [41]. Vetaas and Ferrer-Castán [42] (pp. 1863–1878) mentioned that elevation is one of the most important factors that influence the water and energy regimes in an area. Furthermore, Vetaas et al. [7] (pp. 1652–1663), Bhattarai et al. [20] (pp. 389–400) and Panda et al. [43] (pp. 10850–10860) also reported similar findings that energy and water were the significant factors in determining the pattern of fern and angiosperm species richness across the Himalaya. There is no doubt that the area with abundant energy and water availability favor high species diversity. We consider energy-water conjecture [28] as a possible explanation for the mid-elevation peak of gymnosperms richness in Nepal. Li and Feng [25] (pp. e0140992) also explained that the availability of water has a strong influence on species at low elevation, and the availability of sufficient energy favors the diversity of species at a higher elevation. Thus, the humped-shaped pattern of gymnosperms richness can be better explained by the energy and water, showing the high number of species in the mid-elevation region with sufficient energy and water. Photosynthesis in plants is always favored by available energy and moisture that promotes species richness by influencing all physiological processes [28,31,36,40]. Kluge et al. [24] (pp. 1711–1722) have argued that both above and below the intermediate temperature optimum, plants species richness decline. On the other hand, the result of variation partitioning showed that the combined effect of energy-water and physical tolerance explains the species richness pattern of gymnosperms in Nepal. Energy-water and physical stress both are interdependent to one another and with an increase in elevation, plants are subjected to stress from temperature and water [11,31]. There is evidence that both temperature and precipitation decrease with increasing elevation and the available energy and water to the plants will be limited [3]. Climate and topographic variability restrict the diversity of species at different elevation zones. This restriction might be the effect of physiological limits of the species and the niche differences within which species can survive [11,28,31]. Thus, the strong dependency of gymnosperms on energy-water and physical tolerance variables might be because of the prevailing cold and dry conditions in the area [43]. 

Our findings also supported the physical tolerance hypothesis and that the gymnosperms’ richness has a strong negative relationship with the physical tolerance variables. To our knowledge, this study is the first empirical evidence to provide the linkage between physical tolerance and gymnosperms’ species richness. These results also indicate that an extreme climatic condition causes the abiotic stress to the gymnosperms plants (especially temperature and precipitation). However, these findings contradict the result presented by [44] (pp. 1106–1114). Trisurat et al. [44] (pp. 1106–1114) studied the vulnerability of tree species in the peninsular region of Thailand. The contradictory findings might result because the study was conducted in different plant species rather than gymnosperms and in different climatic conditions between Thailand and Nepal. Trisurat et al. [44] (pp. 1106–1114) considered only trees species in the peninsula region while our study focuses on all growth forms of gymnosperm (tree, shrubs and herbs) in regions that experience climatic variability in highest elevation gradients in the world. On the other hand, similar to our findings, [45] (pp. 568–572) found a significant negative relationship between European plants and the temperature of the warmest month; which indicates that with the increase in temperature during the warm month due to climate change, the diversity of gymnosperms might restrict to a small range or probably shift to the colder range. Climate warming is governing the migration of plant species into higher elevations to avoid regions under climatic stress in search of a more favorable environment [39]. This phenomenon of an upward shift in tree-line of plant species, including gymnosperms due to climate change, has previously been reported in the Himalayan and adjacent regions [39,46]. Moreover, [47] (p. 105559) reported the significant role of the climatic stability of the warmest quarter during the quaternary and current times. This climatic stability was an ecological indicator for the range stability of cold temperate conifers in the high elevations of south-western China, including the eastern part of the Tibetan Plateau. This might be the possible reason for the range shift in gymnosperms across the Himalaya due the change in temperature and precipitation during the driest months.

In this study, a strong and significant negative relationship was found between climatic seasonality predictor set and species richness while the variation partitioning showed the least effect of climatic seasonality compared to the other two predictor sets. Opposite to our findings, a significant positive relationship between vascular plant species richness and precipitation seasonality was reported in China [48]. On the other hand, climatic seasonality was important variable in determining the distribution of *Rhododendron* in China [35]. Tropical and temperate mountains ecosystem experience uniform seasonal variation [35], thus, the least explained variation in species richness pattern of gymnosperms by climatic seasonality might occur due to the physiological barrier that limits the growth of species in a specific area.

## 4. Materials and Methods 

### 4.1. Study Area and Species Data

This study covered the entire area of gymnosperms distribution within the geographical territories of Nepal (26.369 N to 30.425 N latitudes and 80.052 E to 88.194 E longitudes). The distance from north to south of the country is 150–200 km and region exhibits high topographic and climatic variation within a small geographical region. Nepal has the most extended elevation gradient, from 60 m, i.e., the flatland of the south to snow-capped mountains in the north, including the highest mountain peak, The Mount Everest [8848m; [37]]. Dobremez has classified the country based on ecology and biogeography and described 75 vegetation types favored by complex habitat and ecological zones, making it ideal land for plants growth [49]. Thus, the country is rich in biodiversity, including gymnosperms. Nepal is home to 30 taxa of native gymnosperm, grouped into seven taxonomic families, 13 genera, 26 species [50]. In this study, we focused on the range distribution of 26 species of gymnosperms based on elevation and environmental gradients (list of species and its maximum and minimum elevation limit is in Table S2 of Supplementary Materials). We used “The Plant List” portal (http://www.theplantlist.org) to validate the names of all species for synonyms and nomenclature errors, and only the accepted names of the species were used in this study.

The elevation ranges of species distribution were extracted from [51]. However, we referred to Flora of Nepal database (http://www.floraofnepal.org/, accessed between March 2019 and December 2019), herbarium specimens and relevant literature for missing information. From our preliminary study, we found that all the species of gymnosperms in Nepal were distributed between 200 m and 5400 m elevation range. The upper limit is the last elevation range, where the distributions of species of gymnosperms were reported. Following [8] (pp. 25–33), we divided the elevation range of the country from 200 m to 5400 m into a 100 m elevation band; thus, our study comprised of 53 elevational bands.

### 4.2. Species Richness

Species richness denotes the response variable in this study, defined as the number of gymnosperms species present in each elevation band, obtained via interpolation methods [52]. We interpolated the presence of species based on the elevational range of species from its minimum and maximum elevation distribution. This method assumed that taxa were found at all elevations distributed between their lowest and highest limits. The interpolation method helps in overcoming the problem of under-sampling as well. Following [30] (pp. 6872–6879), we performed polynomial regression to determine the relationship between species richness along elevation bands. Moreover, due to the conical shape of the mountains, the surface area of the mountain will be more at the base than the top [3]. The surface area of each elevation band will be different from one another, and with the change in the land area across elevation; species richness varies [53]. The area in each elevation band is also a surrogate variable for the size of the gene pool and can directly influence the species richness [54]. Therefore, to avoid the biased of areas affecting species richness and for the purpose of analyses, following [30] (pp. 6872–6879), we divided the number of species in each elevation band by the surface area of respective elevation interval. These represent the gymnosperms’ species richness that was used for further analysis. We calculated the surface area of each 100 m elevation interval in ArcGIS (v10.3.1) (ESRI Inc., Redlands, California, USA). We used a digital map of the country along with elevation from Digital Elevation Mercator (DEM) and divided the map of the region into the required elevation bands. The data of elevation and area along elevation gradients were downloaded from Shuttle Radar Topography Mission (SRTM; https://www2.jpl.nasa.gov/srtm/) [55].

### 4.3. Predictor Variables

The elevation directly or indirectly determines the gradient in environmental variables, while these environmental gradients have a direct relationship with the growth and development of plants [36]. We used thirteen environmental variables to evaluate the pattern of species richness of gymnosperms along elevation gradients. Based on the objective of studies, we separated these variables into three distinct predictor sets namely- (i) energy-water, (ii) physical tolerance, and (iii) climatic seasonality. 

Previous studies have shown that annual mean temperature (MAT, ºC/year), annual mean potential evapotranspiration (PET, mm/year), mean annual precipitation (MAP, mm/year) and annual mean actual evapotranspiration (AET, mm/year), are the significant surrogate variables representing the source of energy and water [30,35]. MAT and MAP were calculated as the mean annual temperature and mean annual cumulative precipitation, respectively, from the data taken from climatic stations located in 96 different locations within the country. There were no climate stations above 4100 m; thus, we used the interpolation technique to derive the values of MAT and MAP for each elevational zone above 4100 m. PET and AET were downloaded from the MODIS Global Evapotranspiration Project [41]; http://www.ntsg.umt.edu/project/modis/mod16.php). Values of PET and AET were extracted from an image, processed using ArcGIS. 

We used mean maximum temperature of the warmest month (MTWM, ºC/year), minimum temperature of the coldest month (MTCM, ºC/year), precipitation of the wettest month (PWM, mm/year) and precipitation of the driest month (PDM, mm/year) are the extreme environmental factors used as proxy variables of physical tolerance. Gao and Liu [30] and Jiang et al. [56] have confirmed the use of similar variables in determining the species richness of plants. All the surrogate variables of physical tolerance were calculated from the data available from the climatic stations mentioned above. 

Finally, mean diurnal range (MDR, ºC/year), the annual range of temperature (TAR, ºC/year), temperature seasonality (TES) and precipitation seasonality (PES, mm/year) were surrogate variables of climatic seasonality. These variables were also used in previous studies to represent the climatic seasonality [30,35]. Following [57] (pp. 1965–1978) we calculated MTWM, MTCM, PWM, PDM, MDR, TAR, TES and PES from temperature and precipitation data collected from field station. Similar to MAT and MAP, there was no field station above 4100 m, so we used interpolation technique as mentioned above.

### 4.4. Statistical Analyses

All the explanatory variables were highly correlated to one another. Therefore, we performed Principal Component Analysis (PCA) to reduce multicollinearity in a model. PCA was performed in each predictor set, and we extracted three principal components [58]. The PCA components accounted for 99.2% of the variation in energy-water variables, 97.2% physical tolerance and 99.5% in climatic seasonality variables (Table S3 of Supplementary Materials). 

We performed generalized linear models (GLMs) regression with negative binomial regression (NBR) distribution. NBR is a common method intensively used for the over-dispersed count data [35]. We followed the variable selection approach to identify the best-supported model selecting the predictors based on the least Akaike’s information criterion (AIC) value [59,60]. We used adjusted-*R*^2^% (*R*^2^_adj_%) to account for the percentage of variance explained by each predictor set [61]. Variance inflation factor (VIF) was used to check multicollinearity among response variables used in a model [60]. All the variables in the model have VIF less than 5. Furthermore, we tested the residual of GLM models for spatial autocorrelation using the Moran’s *I* correlogram to test the *type I* error [60]. The tests showed a lack of spatial autocorrelation (Figure S2).

Finally, we used variation partitioning to determine the individual and combined fraction of all predictor sets. Variation partitioning allows us to access the pure effects of the predictor sets and their shared contribution in better explaining the species richness pattern of gymnosperms [62]. This technique is commonly used in community ecology when two or more complementary sets of hypotheses possibly determined the variations in species richness [63]. In this study, we determined the effect of three sets of response variables on species richness using *R*^2^-adjusted (*R*^2^_adj_) as the goodness-of-fit. Variation partition led to eight fractions: (a) fraction explained by energy-water, (b) fraction explained by physical tolerance, (c) fraction explained by climatic-seasonality, (d) fraction explained by joint effect of energy-water and physical tolerance, (e) fraction explained by joint effect of physical tolerance and climatic seasonality, (f) fraction explained by joint effect of energy-water and climatic seasonality, and finally, (g) joint fraction between all three predictor sets. The residual is the unexplained fraction of variation partitioning (Figure 3 and Table S1 Supplement Materials). Variation partitioning technique was previously used in explaining the effect of environmental factor on species richness patterns [30,54]. The relationship between species richness and its environmental correlates using GLM and variation partitioning allows us to compare the relative importance of energy-water, physical tolerance, and climatic seasonality in explaining the species richness patterns of gymnosperms in Nepal along elevation gradients.

All the statistical analysis was performed in R statistical packages [64]. PCA was performed using “vegan” package, “MASS” package was used for GLM regression, “car” to check VIF of a model, “var.part” for variation partitioning between response variables and species richness. Digitization and processing of maps were done using ArcGIS.

## 5. Conclusions

The distribution of gymnosperms forms a hump-shaped pattern and strongly correlates to the environmental condition of the area. We have evidence that the unimodal distributional pattern of gymnosperms along elevation gradients was mostly determined by the relative importance of energy-water followed by climatic seasonality and physical tolerance predictor set. The strong correlation between species richness and environmental variables indicated the importance of multi-gradient studies for testing elevation richness pattern. Besides, the important effect of environmental variables and the response of gymnosperms towards the differential intensity of changing climatic variables suggested that our study might enhance understanding of the response of gymnosperms to climate change. The event of global warming and climate change pattern might affect the montane biota and will likely cause a shift in range distribution. This study serves as a guideline for ecologists and policymakers in designing conservation plans for the management of protected areas and will enable readers to understand the species richness patterns of gymnosperms along elevation gradients in Nepal. However, taking the findings of the present study, detailed research is required to access the conservation status of gymnosperms in Nepal. Moreover, future studies should also focus on the prediction of distribution pattern of gymnosperms under different climate change scenarios and how they respond in changing climatic conditions. 

## Figures and Tables

**Table 1:** Percentage of coefficient of determination (*R^2^_adj_*, %) by the generalized linear models (GLMs) between species richness of gymnosperms and predictor variables sets representing each hypothesis set. *p*-value is the significance value and AIC is the Akaike’s information criterion value of each model. Numbers in parentheses are the coefficient of respective variables.

Hypotheses	Predictor Variables Included in The Best Model (Coefficient of Variables)	Percentage of Coefficient of Determination (% of *R*^2^_adj_)	*p*-Value	AIC
Energy-Water	EW1 (−0.2968) ***	77.93	<0.001	219.9
	EW2 (+0.4821) ***			
	EW3 (−0.6361) ***			
Physical tolerance	PT1 (−0.2762) **	38.64	<0.001	297.2
	PT2 (−0.4276) ***			
Climatic Seasonality	CS2 (−0.6052) ***	46.80	<0.001	289.1
	CS3 (−0.1697) *			

EW, PT and CS refer to variables based on the first three axes of the principal components analysis (PCA) using energy-water, physical tolerance and climatic seasonality variables, respectively. Significance levels of each variable in the model are * p < 0.05, ** p < 0.01, *** *p* < 0.001.

## Supplementary Materials

The following are available online at https://www.mdpi.com/2223-7747/9/5/625/s1, Figure S1: Relationship between environment variables along elevation gradients in Nepal, Figure S2: Moran’s Index correlograms for gymnosperms richness and residuals autocorrelation, Table S1: Results of variation partitioning showing the percentage contribution of predictor variables to determine the species richness of gymnosperms along elevation gradients, Table S2: List of species of gymnosperms reported from Nepal along with the lower and upper elevational limits expressed in meter (m) above sea level, Table S3: Results of principal components analysis using energy-water, physical tolerance and climatic seasonality sets of variables.
