# Supplementary material for: Distribution Pattern of Gymnosperms’ Richness in Nepal: Effect of Environmental Constrains along Elevational Gradients
Source: Plants (Basel). 2020 May 14;9(5):625. doi: 10.3390/plants9050625 (PMC7285339; doi:10.3390/plants9050625)
Supplement: Supplementary file 1 [file plants-09-00625-s001.pdf]

Figure S1. Relationship between environment variables along elevation gradients in Nepal.

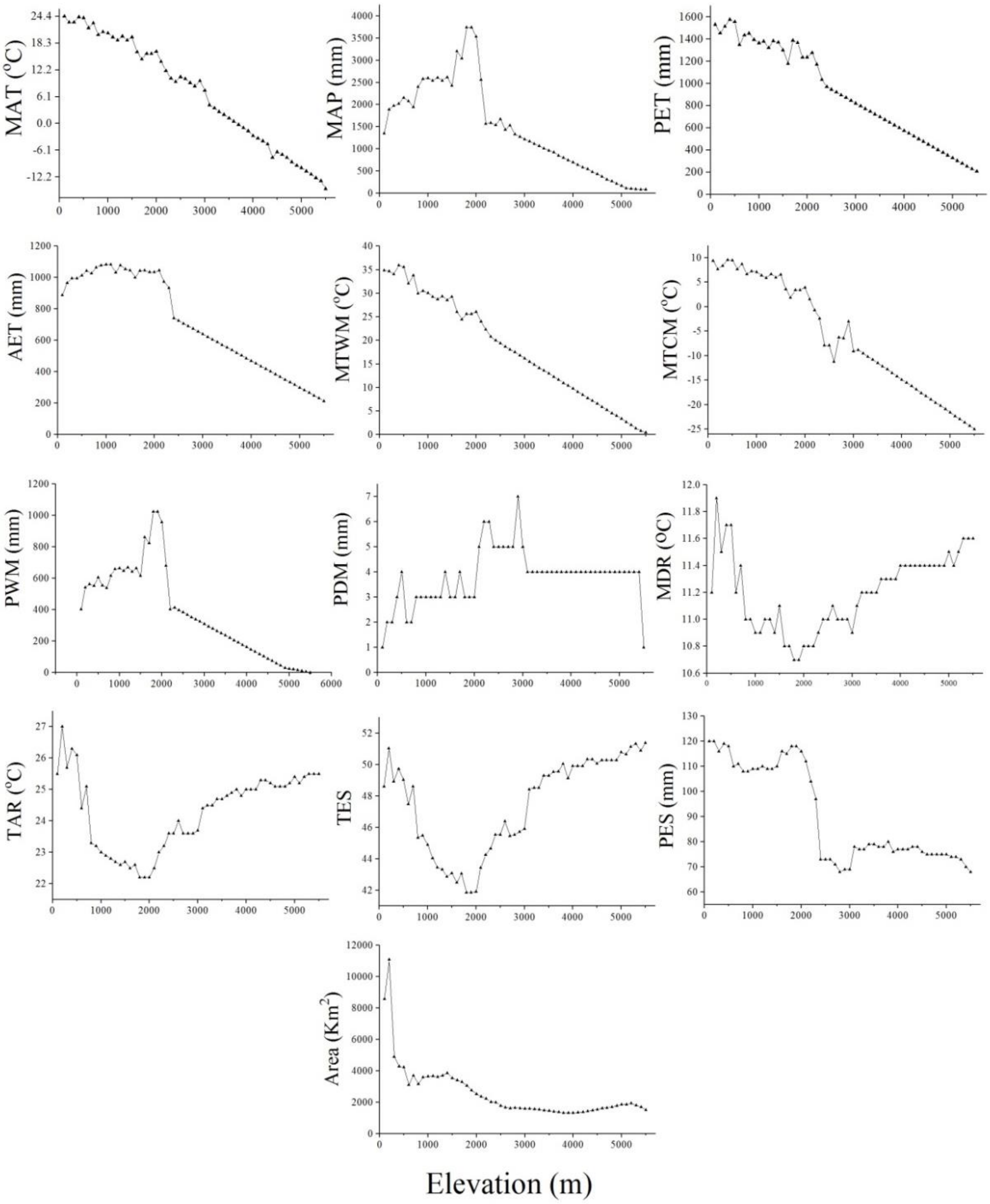

MAT, Mean Annual Temperature; MAP, Mean Annual Precipitation; PET, Potential Evapotranspiration; AET, Actual Evapotranspiration; MTWM, Maximum temperature of the warmest month; MTCM, Minimum temperature of the coldest month; PWM, precipitation of the wettest month; PDM, precipitation of the driest month; TES, Temperature Seasonality; PES, Precipitation Seasonality; TAR, Annual Range of Temperature.

Figure S2. Moran's Index correlograms for gymnosperms richness and residuals autocorrelation.

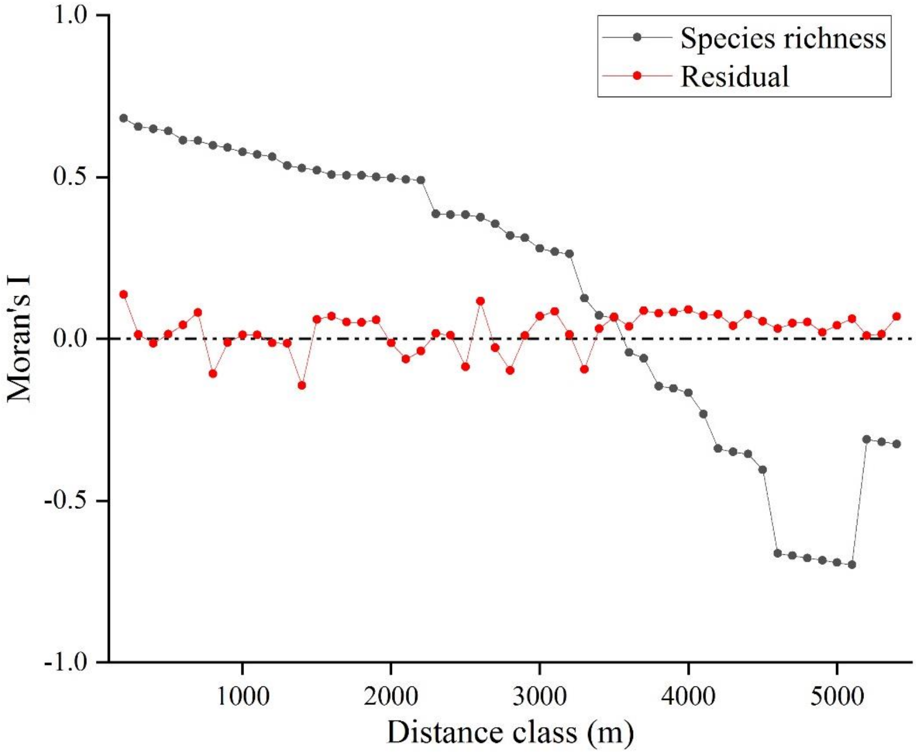

Table S1. Results of variation partitioning showing the percentage contribution of predictor variables to determine the species richness of gymnosperms along elevation gradients. The lowercase letter denotes the individual contribution (adjusted R^2) following the labels displayed in figure 3 (main text).

Individual contribution (%)	Species richness
[a]	24.62
[b]	7.9
[c]	1.15
[d]	29.62
[e]	5.28
[f]	27.72
[g]	2.62
[h] = Residuals	8.17
All environmental variables [abcdefg]	91.82
Total energy-water set [adfg]	84.59
Total physical tolerance set [bdeg]	38.32
Total climatic seasonality set [cefg]	36.78

Table S2. List of species of gymnosperms reported from Nepal along with the lower and upper elevational limits expressed in meter (m) above sea level.

Name of Species	Lower limit (m)	Upper limit (m)
Abies densa Griff.	3000	4000
Abies pindrow (Royle ex. D.Don) Royle	2100	2500
Abies spectabilis (D. Don) Mirb.	2400	4400
Cedrus deodara (Royle ex. D.Don) G.Don	2000	2500
Cupressus torulosa D. Don	2000	4600
Cycas pectinata Buch.-Ham.	300	450
Cycas revoluta Thunb.	400	700
Ephedra gerardiana Wall. ex Stapf	3700	5300
Ephedra intermedia Schrenk & C.A. Mey	2000	4600
Ephedra pachyclada Boiss.	2700	4100
Gnetum montanum Markgr.	300	1800
Juniperus communis L.	2700	5000
Juniperus indica Bertol.	3700	4100
Juniperus recurva Buch.-Ham. ex. D.Don	3300	4600
Juniperus squamata Buch.-Ham. ex. D.Don	3300	4400
Larix griffithii Hook.f.	1400	3900
Larix himalaica W.C. Cheng & L.K. Fu	2400	3600
Picea smithiana (Wall.) Boiss.	2300	4300
Picea spinulosa (Griff.) A. Henry	2300	3600
Pinus roxburghii Sarg.	1100	2100
Pinus wallichiana A.B. Jacks	1800	3300
Podocarpus neriifolius D.Don	1000	1400
Taxus contorta Griff.	2200	4400
Taxus mairei (Lemée & H.Lév.) S.Y.Hu	1600	2400
Taxus wallichiana Zucc.	1600	3500
Tsuga dumosa (D. Don) Eichler	2100	3600

Table S3. Results of principal components analysis using energy-water, physical tolerance and climatic seasonality sets of variables.

**Energy-water**	**PC1**	**PC2**	**PC3**
MAT	0.967	-0.118	0.189
MAP	0.941	0.333	-0.012
AET	0.985	0.032	0.149
PET	0.983	-0.100	0.139
MAT^2	0.830	-0.445	-0.335
MAP^2	0.844	0.479	-0.239
AET^2	0.989	0.013	0.028
PET^2	0.977	-0.186	-0.003
Proportion of variance (%)	88.6	7.5	3.1
Cumulative Proportion (%)	88.6	96.1	99.2
**Physical tolerance**	**PC1**	**PC2**	**PC3**
MTWM	0.175	-0.082	0.430
MTCM	0.177	-0.098	0.300
PWM	0.172	-0.094	-0.539
PDM	-0.104	-0.461	-0.086
$MTWM^2$	0.171	0.012	0.539
$MTCM^2$	-0.142	0.294	-0.126
PWM^2	0.154	-0.020	-0.989
PDM^2	-0.087	-0.491	0.030
Proportion of variance (%)	68.4	22.1	6.7
Cumulative Proportion (%)	68.4	90.9	97.2
**Climatic seasonality**	**PC1**	**PC2**	**PC3**
MDR	0.151	0.187	1.652
TAR	0.156	0.119	-0.326
TES	0.159	-0.020	-1.648
PES	-0.09	0.501	-0.723
MDR^2	0.150	0.192	1.672
TAR^2	0.156	0.130	-0.263
TES^2	0.159	-0.016	-1.625
PES^2	-0.090	0.503	-0.502
Proportion of variance (%)	77.8	20.6	1.1
Cumulative Proportion (%)	77.8	98.4	99.5

MAT, Mean Annual Temperature; MAP, Mean Annual Precipitation; PET, Potential Evapotranspiration; AET, Actual Evapotranspiration; MTWM, Maximum temperature of the warmest month; MTCM, Minimum temperature of the coldest month; PWM, precipitation of the wettest month; PDM, precipitation of the driest month; TES, Temperature Seasonality; PES, Precipitation Seasonality; TAR, Annual Range of Temperature.
